# Non-randomized therapy trial to determine the safety and efficacy of heavy ion radiotherapy in patients with non-resectable osteosarcoma

**DOI:** 10.1186/1471-2407-10-96

**Published:** 2010-03-12

**Authors:** Claudia Blattmann, Susanne Oertel, Daniela Schulz-Ertner, Stefan Rieken, Sabine Haufe, Volker Ewerbeck, Andreas Unterberg, Irini Karapanagiotou-Schenkel, Stephanie E Combs, Anna Nikoghosyan, Marc Bischof, Oliver Jäkel, Peter Huber, Andreas E Kulozik, Jürgen Debus

**Affiliations:** 1 Department of Pediatric Oncology, Hematology and Immunology, University of Heidelberg, Im Neuenheimer Feld 154, 69120 Heidelberg, Germany; 2 Department of Radiotherapy, University of Heidelberg, Im Neuenheimer Feld 400, 69120 Heidelberg, Germany; 3 Markus Krankenhaus, Frankfurter Diakonie-Kliniken, Wilhelm Epstein-Strasse 4, 60431 Frankfurt am Main, Germany; 4 Department of Nuclear Medicine, University of Heidelberg, Im Neuenheimer Feld 400, 69120 Heidelberg, Germany; 5 Department of Orthopedic Surgery, University of Heidelberg, Schlierbacher Landstrasse 200a, 69118 Heidelberg, Germany; 6 Department of Neurosurgery, University of Heidelberg, Im Neuenheimer Feld 400, 69120 Heidelberg, Germany; 7 National Centre for Tumor Diseases (NCT) Heidelberg, Im Neuenheimer Feld 581, 69120 Heidelberg, Germany

## Abstract

**Background:**

Osteosarcoma is the most common primary malignant bone tumor in children and adolescents. For effective treatment, local control of the tumor is absolutely critical, because the chances of long term survival are <10% and might effectively approach zero if a complete surgical resection of the tumor is not possible. Up to date there is no curative treatment protocol for patients with non-resectable osteosarcomas, who are excluded from current osteosarcoma trials, e.g. *EURAMOS1*. Local photon radiotherapy has previously been used in small series and in an uncontrolled, highly individualized fashion, which, however, documented that high dose radiotherapy can, in principle, be used to achieve local control. Generally the radiation dose that is necessary for a curative approach can hardly be achieved with conventional photon radiotherapy in patients with non-resectable tumors that are usually located near radiosensitive critical organs such as the brain, the spine or the pelvis. In these cases particle Radiotherapy (proton therapy (PT)/heavy ion therapy (HIT) may offer a promising new alternative. Moreover, compared with photons, heavy ion beams provide a higher physical selectivity because of their finite depth coverage in tissue. They achieve a higher relative biological effectiveness. Phase I/II dose escalation studies of HIT in adults with non-resectable bone and soft tissue sarcomas have already shown favorable results.

**Methods/Design:**

This is a monocenter, single-arm study for patients ≥ 6 years of age with non-resectable osteosarcoma. Desired target dose is 60-66 Cobalt Gray Equivalent (Gy E) with 45 Gy PT (proton therapy) and a carbon ion boost of 15-21 GyE. Weekly fractionation of 5-6 × 3 Gy E is used. PT/HIT will be administered exclusively at the Ion Radiotherapy Center in Heidelberg. Furthermore, FDG-PET imaging characteristics of non-resectable osteosarcoma before and after PT/HIT will be investigated prospectively. Systemic disease before and after PT/HIT is targeted by standard chemotherapy protocols and is not part of this trial.

**Discussion:**

The primary objectives of this trial are the determination of feasibility and toxicity of HIT. Secondary objectives are tumor response, disease free survival and overall survival. The aim is to improve outcome for patients with non-resectable osteosarcoma.

**Trail Registration:**

Registration number (ClinicalTrials.gov): NCT01005043

## Background

Osteosarcoma is the most common primary malignant bone tumor in children and adolescents but also occurs in adults. The most frequent primary tumor sites are the distal femur and the proximal tibia, but there are a significant number of patients with axial primary tumors or other unfavorable, non-resectable tumor sites (5-10%). Effective treatment depends on neoadjuvant and adjuvant chemotherapy to control micrometastatic disease, and on radical surgical resection to control the primary tumor. Local control of the tumor is absolutely critical, because the chances of long term survival are <10%, if a complete surgical resection of the tumor is not possible [1,2]. In a minority of patients, the tumor cannot be completely resected without unacceptable mutilation. In such patients local radiotherapy has previously been used. Published small series of such patients have documented that radiotherapy can achieve local control [3], although the success rate of this strategy has not been analyzed in systematic trials.

Although the small series of patients with non-resectable tumors, who received local radiotherapy but no surgery showed encouraging results, the necessary high radiation doses cannot be achieved in many patients with tumors near critical organs such as the brain, the spinal cord or small bowel. In such cases PT plus HIT may offer a promising alternative.

Compared with photons, heavy ion particles such as carbon ions or protons provide a higher physical selectivity because of their finite depth coverage in tissue and, in the case of heavy ions, achieve a higher relative biological effectiveness. Phase I/II dose escalation studies of HIT in adults with non-resectable bone and soft tissue sarcomas showed favorable results [4,5]. Kamada et al. demonstrated a 3-year overall survival rate of 45% in a series of 15 patients with non-resectable osteosarcoma of the pelvis (10 patients) or the spine (5 patients). These results are clearly superior to most published 3-year survival rates in patients with non-resectable osteosarcoma of the pelvis and/or spine, who did not receive radiotherapy [6,7].

Since 1997, HIT is available for the treatment of patients at the Gesellschaft für Schwerionenforschung (GSI) in Darmstadt, Germany. Up to now, more than 400 patients with various tumor types were successfully irradiated at the GSI [8,9]. From November 2009, HIT and PT will be available at the University of Heidelberg. In contrast to other centers, the facility at Heidelberg relies on active beam delivery using the so called raster scan technique, which offers physical advantages with better sparing of normal tissue in the entrance channel, less scattering dose. Furthermore, biological plan optimization is performed.

This study aims at improving outcome in patients with non-resectable osteosarcoma

## Methods/Design

### Study design

The study is designed as a mono center, single arm phase I/II pilot trial.

### Study objectives

The primary objective is to evaluate the feasibility and toxicity of dose escalation via PT/HIT in patients with osteosarcoma. Secondary objectives are the evaluation of tumor response, disease free survival, overall survival and description of FDG-PET characteristics in patients with osteosarcoma before and after PT/HIT in order to evaluate prospectively the association between FDG-PET and survival respectively time to progression.

### Trial organization

The trial has been designed by the study initiators at the Department of Radiation Oncology and the Department of Pediatric Oncology, Hematology, Immunology and Pneumology. The trial is carried out by the Department of Radiation Oncology and the Department of Pediatric Oncology, Hematology, Immunology and Pneumology at the University of Heidelberg.

### Coordination

The overall coordination is performed by the Department of Radiation Oncology at the University of Heidelberg. This department is responsible for the overall trial management, database management, quality assurance including monitoring and reporting.

### Investigators

The study investigators are experienced radiation oncologists respectively pediatricians. Patients will be treated by the physicians of the Department of Radiation Oncology and the Department of Pediatric Oncology, Hematology, Immunology and Pneumology.

### Ethics, informed consent and safety

The final protocol was approved by the ethics committee of the University of Heidelberg, Heidelberg, Germany (Nr. S-153/2008). This trial complies with the Helsinki Declaration in its recent German vision, the medical Association code of conduct, the principles of Good Clinical Practice (GCP) and the Federal Data Protection Act. The study will also be carried out in keeping with local legal and regulatory requirements. It is subjected to authorization by the "Bundesamt für Strahlenschutz". The medical secrecy and the Federal Data Protection Act will be followed.

### Patient selection

The following inclusion criteria must be fulfilled:

▪ Histological diagnosis of high grade osteosarcoma with or without metastases

▪ Non-resectable* tumor of the pelvis, the skull base or the spine, respectively, incomplete or intralesional tumor resection - as confirmed after evaluation by two orthopedic surgeons (respectively neurosurgeons in case of spine tumors): one local surgeon and one referee surgeon of the University of Heidelberg.

▪ Age ≥ 6 years before start of radiotherapy

▪ Adequate performance status (Karnofsky ≥ 60%)

▪ Adequate blood cell production before the start of PT/HIT in patients with pelvic or spine tumors as defined by: total white cell count (WBC) ≥ 1,0/nl; neurophils ≥ 200/μl; platelet count ≥ 20/nl

▪ No febrile neutropenia (neutrophils < 200/μl)

▪ Written informed consent of the patient or the legal guardians

*Non-resectable tumor site means primary tumors affecting anatomic areas of the human body where a surgical total resection (R0) of the tumor is not possible for technical reasons, for example osteosarcoma of the pelvis, spine or the skull base. In any other cases, surgical resection is recommended.

Non-resectability has to be confirmed after evaluation by two orthopedic surgeons (respectively neurosurgeons in case of spine tumors): one local surgeon and one referee surgeon of the University of Heidelberg.

In some cases surgery of the tumor might be possible after PT/HIT. Than we recommend surgical resection of residual tumor afterwards.

### Exclusion criteria

▪ Age < 6 years

▪ Previous radiotherapy of the field that has to be radiated now

▪ Implanted metal within the planned radiation field, that leads to significant artefacts within the target volume

▪ Patients receiving any other investigational agents during the time of HIT

▪ Performance status (Karnofsky) ≤ 60%)

▪ Pregnancy

▪ No written informed consent of patient or the legal guardians

### Statistical calculations for trial sample size

20 patients ≥ 6 years will be included in the trial. This number ensures acceptable 95%-confidence intervals for the description of incidence of toxicities as well as for the efficacy of the HIT-treatment in this trial. For response rates of 30% the 95%-confidence interval will be 17.9-44.6%.

### Adverse events

Radiotherapy-related toxicities will be assessed using the NCI Common Terminology Criteria (CTC). Toxicity will be evaluated pre-treatment, weekly during radiation therapy (blood count, electrolytes, chemistry, clinical examination, patient visits) and at follow-up (1 week, 6 and 19 weeks, 6, 12, 24, 36 48 and 60 months after PT/HIT). Unacceptable toxicity is defined as unpredictable or irreversible grade 4 toxicity.

Expectable possible acute toxicities (up to 3 months post PT/HIT) are

- Depression of hematopoesis

- Mucosa: ulcera, chronic inflammation, colic, diarrhoea, bowel stenosis, urinary bladder inflammation, contracted bladder

- Skin: erythema, epitheliolysis

- CNS: sickness, vomiting, central nerve palsy, elevated intracranial pressure, headach, dizziness

- sceletal system: necrosis, fracture, joint dysfunction, disturbance of growth

Late effects are rare and defined as symptoms appearing at least 3 months post irradiation. These could include:

- Bowel stenosis or perforation with need for surgical intervention

- Chronic bowel inflammation

- CNS: central nerve palsy, cerebral palsy, impaired vision, defective hearing (very rare)

- Disturbance of growth

- Secondary malignancies: The incidence of secondary malignancies is possibly increased by high-LET radiotherapy like HIT

- arteriosclerosis

- Incontinence (very rare)

- Impotence (very rare)

A rate of acute toxicity (≤ 3 months during respectively after PT/HIT) > grade 3 of ≤ 5% and a rate of late toxicity (after ≥ 3 months) > grade 3 of ≤ 3% will be regarded as acceptable.

### Radiation therapy, process of PT/HIT

All patients with osteosarcoma who meet the above mentioned criteria will be included. Chemotherapy prior and after HIT is recommended according to standard therapy protocols like EURAMOS1 and is not part of this trial protocol.

PT and HIT will be administered at the Department of Radiotherapy of Heidelberg University at least one week after the last chemotherapy cycle, if the inclusion criteria have been met. HIT will be given mainly on an outpatient basis on 5-6 days per week but with daily clinical examinations by a radiotherapist respectively by a pediatrician. Necessity of inpatient basis has to be verified individually for example in case of adverse events during PT/HIT. Then the treatment will be arranged and supervised by the Department of Pediatric Oncology, Hematology, Immunology and Pneumology of the Heidelberg University.

No treatment other than PT/HIT may be administered during PT/HIT.

**Figure 1:**
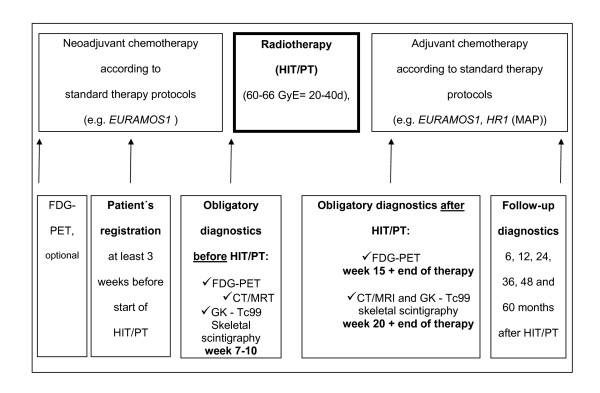
**Chronological course of the therapy trial**. HIT/PT will be administered at least one week after the last chemotherapy cycle on 6 days per week. Chemotherapy prior and after HIT/PT is recommended according to standard therapy protocols and is not part of this trial protocol. After the end of HIT/PT, adjuvant chemotherapy can be continued within one week. Diagnostic investigations have to be done at a specific date pre and after HIT/PT. FDG-PET characteristics will be evaluated prospectively.

After the end of PT/HIT, adjuvant chemotherapy can be continued within one week according to standard therapy protocols, e.g. according to EURAMOS 1 trial arm "poor response" (figure 1).

### Technique

Patients will be rigidly immobilized to ensure a high repositioning accuracy of the tumor region and adjacent structures for PT/HIT. Set-up deviations larger than > 1 mm in the skull base region and larger than 3 mm in the spinal and pelvic region will be corrected prior to each irradiation. If necessary, children will be anesthetized for the procedure.

The planning target volume results from the clinical target volume plus a safety margin that depends on tumor site and immobilization technique.

GTV (Gross Tumour Volume) is delineated according to MRI/CT-Scan/PET-CT.

CTV(Clinical Target Volume) contains the visible GTV + the subclinical microscopic malignant disease. The original (pre-chemotherapy, pre-operative) tumour extension should guide the CTV-delineation. In axial tumours a clinical safety margin of 2 cm added to the GTV should be attempted. For extremity sarcomas a margin of 4-5 cm around the GTV is preferred.

PTV (Planning Target Volume) is delineated in collaboration with the radiation-physicist taking into account organ and patient movement as well as inaccuracies in beam and patient set-up.

Organs at risk such as rectum, bladder, femoral heads, central nerves, spinal cord are contoured.

The quality of patient immobilization will be checked quantitatively daily via digital x-ray equipment. During HIT, set-up is checked daily with a system based on orthogonal x-ray devices and set-up deviations >1 mm for skull base tumors and >3 mm for pelvic/spinal/extremity tumors are corrected prior to PT/HIT.

### Treatment planning

Treatment planning includes biological plan optimization using the local effect model (LEM), which is included into the treatment planning software. Active beam delivery using the raster scan system is used to spare normal tissue surrounding the tumor. Desired target dose is 60-66 Cobalt Gray Equivalent (Gy E), whenever possible. It is applied through 1 - 3 isocentric treatment portals via proton therapy plus a heavy ion boost of 15-21 GyE. Dose distributions are calculated and dose volume histograms (DVH) are generated. A α/β-ratio of 2 is used for biological plan optimization. Fractionation is planned to be equivalent to 5 × 2 Gy PT per week and 5-6×/3 Gy E HIT per week. Dosage to organs at risk is minimized.

### Duration of Therapy

Treatment continues for **20 to 40 days **or until one of the following criteria applies:

▪ Other illness that prevents further administration of treatment,

▪ Patient or legal guardian decides to withdraw from the study, or

▪ General or specific changes in the patient's condition render the patient unacceptable for further treatment in the judgment of the investigator.

Because the tumors of the patients who are eligible to be included in this study will not be resectable, the regression grade following chemotherapy cannot be determined according to standard criteria. A recent study showed significant alterations of glucose-uptake in FDG-PET of osteosarcoma after response to chemotherapy, which correlated with the histopathologic assessment of response [10-12]. Therefore, in this study FDG-PET will be used for the prospective evaluation of tumor response after PT/HIT. If possible, FDG-PET will be performed before start of neoadjuvant chemotherapy. FDG-PET has to be performed obligatory no more than one week before the initiation of PT/HIT (week 10), after PT/HIT (week 15), at the end of the complete tumor therapy (week 30) and 6 months after tumor therapy (figure 1).

### Measurement of response

Response and progression will be evaluated in this study using the new international criteria proposed by the Response Evaluation Criteria in Solid Tumors (RECIST) Committee [13]. Changes in the largest diameter (one-dimensional measurement) of the tumor lesions are used in the RECIST criteria.

#### Prospective Evaluation of Fluorodeoxy-D-Glucose Positron Emission Tomography (PET)

A secondary objective of this study is to describe the FDG-PET imaging characteristics in patients with osteosarcoma before and after HIT and to determine the association between FDG-PET and survival respectively time to progression.

FDG-PET has to be performed obligatory at 4 time points: one week before start of PT/HIT, after PT/HIT, at the end of complete tumor therapy and 6 months after PT/HIT. If possible, FDG-PET has to be performed initially before start of neoadjuvant therapy.

Results, respectively response criteria will be evaluated in future.

### Time plan for the study

The study will be started in January 2010. Patient recruitment will be completed when 20 patients are included which is expected latest in 2015. Patient follow-up will continue for at least 5 years within our center, and will continue on a regular written basis (once yearly). The trial will be completed latest in 2020.

### Follow-up

Follow-up visits are obligatory 1 week, 6 and 19 weeks, as well as 6, 12, 24, 36 48 and 60 months after PT/HIT. For a detailed plan of follow-up diagnostics see table 1.

**Table 1:** Obligatory Assessment after HIT/PT

	Visit Department of Radiooncology, Heidelberg	Physical Examinations and Blood Tests	Chest X-ray	Chest CT-Scan	X-ray of Primary Tumor Site or Skeletal Metastases	CT/MRI of Primary Tumor Site or Skeletal Metastases	Whole Body FDG-PET	Whole Body Tc99 bone scan
Week 14	X	X					X	
Week 19	X	X	X	If chest x-ray shows strong suspicion of metastases	X	X		X
Week 33	X	X	X	If chest x-ray shows strong suspicion of metastases	X	X	X	X
6 months after PT/HIT	X	X	X	If chest x-ray shows strong suspicion of metastases	on clinical suspicion of metastases	X	X	on clinical suspicion of metastases
12 months after PT/HIT	X	X	X	If chest x-ray shows strong suspicion of metastases	on clinical suspicion of metastases	X	on clinical suspicion of metastases	on clinical suspicion of metastases
24, 36, 48 and 60 months after PT/HIT	X	X	X	If chest x-ray shows strong suspicion of metastases	on clinical suspicion of metastases	X	on clinical suspicion of metastases	on clinical suspicion of metastases

### Monitoring

Monitoring is performed according to good clinical practice (GCP) guidelines. The monitoring and the data management will be performed by the Clinical Trial center of the Department of Radiation Oncology, University of Heidelberg.

## Discussion

Osteosarcoma is the most common bone tumor in children. Local control is absolutely critical concerning survival. Standard therapy protocols consist of surgical resection of the primary tumor and polychemotherapy. Until now, there has been no standard therapy protocol for non-resectable tumors.

For a long time, osteosarcoma was supposed to be a radioresistant tumor, but published small series of osteosarcoma patients have shown that radiotherapy with photons can achieve local control. However, a high radiation dose has to be applied, that usually cannot be given in non-resectable (mainly axial) tumors that are located close to critical organs like the brain, the spine or the bowels.

Particle therapy, respectively proton and carbon ion beams, have the advantage of a superior biological dose distribution that permits radiation with high doses even in the vicinity of critical organs. There is a steep dose fall-off after the so called "Bragg peak", which minimizes effects to normal tissue behind the Bragg peak. Furthermore, the relative biological effectiveness (RBE) of heavy ion beams within the Bragg peak is higher than that of photon beams and therefore has the potential advantage of providing a higher local effect in tumors that, like sarcomas, show low sensitivity to conventional radiation. Promising results have been obtained with HIT for a number of tumors in Japan as well as at the "Gesellschaft für Schwerionenforschung" (GSI) in Darmstadt, Germany. 3 year-local control rates of 73% for bone and soft tissue sarcomas were reported from the National Institute of Radiological Sciences in Japan [4,14]. At the GSI, high local control rates with minimal toxicity have been achieved for patients with chordomas and chondrosarcomas of the skull-base as well as adenoidcystic tumors [8]. From 2009, HIT will also be available at the University of Heidelberg. In contrast to other centres, the facility in Darmstadt (GSI) as well as the new facility in Heidelberg relies on active beam delivery using the so called raster scan technique, which offers physical advantages with better sparing of normal tissue in the entrance channel as well as less scattering dose. Moreover, the treatment planning procedure also includes biological plan optimization [15].

PT plus HIT seems to offer a new promising therapy option in patients with osteosarcoma little is known about side effects of HIT in children, but until now there is no curative alternative for inoperable osteosarcoma. Therefore, we have designed a phase I/II trial for patients with non-resectable osteosarcoma respectively patients who or whose legal guardians refuse local surgical resection. The purpose is to investigate the safety and toxicity of dose escalation via HIT as well as its efficacy in order to improve local control and outcome in these patients.

## Abbreviations

GYE: Cobalt Gray Equivalent; COSS: Cooperative Osteosarcoma Study; CT: Computertomography; CTC: Common Toxicity Criteria; DVH: Dose Volume Histogramm; EURAMOS: European American Osteosarcoma Study; FDG-PET: Fluorodeoxy-D-Glucose Positron Emission Tomography; HIT: Heavy Ion Therapy; LEM: Local Effect Model; MRI: Magnet Resonance Imaging; RBE: Relative Biological Effectiveness; RECIST: Response Evaluation Criteria in Solid Tumors

## Competing interests

The authors declare that they have no competing interests.

## Authors' contributions

CB, SO, SR, DS-E, AK and JD planned, organized, conduct the study and provide medical care and follow-up. SH is an expert in nuclear medicine and gave input into development of FDG-PET evaluation. VE is reference orthopedic surgeon, AU reference neurosurgeon. IS performed statistical planning of the trial protocol. SC, AN, OJ and PH are involved in planning and management of HIT. All authors have read and approved the final manuscript.

## Pre-publication history

The pre-publication history for this paper can be accessed here:

http://www.biomedcentral.com/1471-2407/10/96/prepub

## References

[B1] Bielack S Wulff B Delling G Gobel U Kotz R Ritter J Winkler K Osteosarcoma of the trunk treated by multimodal therapy: experience of the Cooperative Osteosarcoma study group (COSS) Med Pediatr Oncol 1995 24 6 12 10.1002/mpo.2950240103 7968796

[B2] Flege S Kuhlen M Paulussen M Bielack S Juergens H Surgery of primary malignant bone tumors Orthopäde 2003 32 940 8 10.1007/s00132-003-0555-6 14615843

[B3] Oya N Kokubo M Mizowaki T Shibamoto Nagata Y Sasai K Nishimura Y Tsuboyama T Toquchida J Nakamura T Hiraoka M Definitive intraoperative very high dose radiotherapy for localized osteosarcoma in the extrimities Int J Radiat Oncol Biol Phys 2001 51 51 87 10.1016/s0360-3016(01)01603-0 11516856

[B4] Kamada T Tsujii H Tsuji H Yanagi T Mizoe JE Miyamoto T Kato H Yamada S Morita S Yoshikawa K Kandatsu S Tateishi A Efficacy and safety of carbon ion radiotherapy in bone and soft tissue sarcomas J Clin Oncol 2002 20 4466 71 10.1200/JCO.2002.10.050 12431970

[B5] Zhang H Yoshikawa K Tamura K Tomemori T Sagou K Tian M Kandatsu S Kamada T Tsuji H Suhara T Suzuki K Tanada S Tsujii H [(11)C]methionine positron emission tomography and survival in patients with bone and soft tissue sarcomas treated by carbon ion radiotherapy Clin Cancer Res 2004 10 1764 72 10.1158/1078-0432.CCR-0190-3 15014030

[B6] Grimer RJ Carter SR Tillman RM Spooner D Mangham DC Kabukcuoglu Y Osteosarcoma of the pelvis J Bone Joint Surg Br 1999 81 796 802 10.1302/0301-620X.81B5.9241 10530839

[B7] Kawai A Huvos AG Meyers PA Healey JH Osteosarcoma of the pelvis. Oncologic results of 40 patients Clin Orthop Relat Res 1998 348 196 207 10.1097/00003086-199803000-00030 9553553

[B8] Schulz-Ertner D Nikoghosyan A Didinger B Debus J Carbon ion radiation therapy for chordomas and low grade chondrosarcomas--current status of the clinical trials at GSI Radiother Oncol 2004 73 Suppl 2 53 56 10.1016/S0167-8140(04)80014-8 15971310

[B9] Schulz-Ertner D Karger CP Feuerhake A Nikoghosyan A Combs SE Jakel O Edler L Scholz M Debus J Effectiveness of carbon ion radiotherapy in the treatment of skull-base chordomas Int J Radiat Oncol Biol Phys 2007 68 449 57 17363188 10.1016/j.ijrobp.2006.12.059

[B10] Hawkins DS Rajendran JG Conrad EU Bruckner JD Eary JF Evaluation of chemotherapy response in pediatric bone sarcomas by [F-18]-fluorodeoxy-D-glucose positron emission tomography Cancer 2002 94 3277 84 10.1002/cncr.10599 12115361

[B11] Eary JF Conrad EU Bruckner JD Folpe A Hunt KJ Mankoff DA Howlett AT Quantitative (F-19) fluorodeoxyglucose positron emission tomography in pretreatment evaluation and grading of sarcoma Clin Cancer Res 1998 4 1215 1220 9607579

[B12] Folpe AL Lyles RH Sprouse JT Conrad EU Eary JF (F-18) fluorodeoxyglucose positron emission tomography as a predictor pathologic grade and other prognostic variables in bone and soft tissue sarcoma Clin Cancer Res 2000 6 1279 1287 10778952

[B13] Gehan EA Tefft MC Will there be resistance to the RECIST (Response Evaluation Criteria in Solid Tumors)? J Natl Cancer Inst 2000 92 179 81 10.1093/jnci/92.3.179 10655425

[B14] Tsujiih Mizoe J Kamada T Baba B Tsuji h Katoh S Yamada S Yasuda S Ohno T Yanagi T Imair Kagei K Kato H Hara R Hasegawa A Nakajima M Suganen Tamakin Takagir Kandatsu S Yoshikaw K Kishomoto R Miyamoto T Clinical Results of Carbon Ion Radiotherapy at NIRS J Radiation Research 2007 48 Suppl A A1 A13 10.1269/jrr.48.A1 17513896

[B15] Jäkel O Schulz-Ertner D Debus J Specifying Carbon Ion Doses for Radiotherapy: the Heidelberg Approach J Radiat Res 2007 48 Suppl A 87 95 10.1269/jrr.48.A87 17513904

